# Olfactory training in specific anosmia to androstenone and its association with genetic variations of OR7D4

**DOI:** 10.1016/j.ibneur.2025.06.017

**Published:** 2025-07-03

**Authors:** Antonia Gillmeister, Maira Harume Nagai, Christian Margot, Priyanka Meesa, Hiroaki Matsunami, Thomas Hummel

**Affiliations:** a Smell and Taste Clinic, Department of Otorhinolaryngology, University of Dresden Medical School, Fetscherstr. 74, Dresden 01307, Germany; b Columbia Center for Human Development, Department of Medicine, Columbia University Irving Medical Center, 650 West 168th Street, New York, NY 10032, USA; c Department of Psychology, University of Geneva, Switzerland; d Georgetown University School of Medicine, USA; e Department of Molecular Genetics and Microbiology, Duke University School of Medicine, 213 Research Dr., Durham, NC 27710, USA

**Keywords:** Androstenone, Olfactory training, OR7D4, Olfaction, Anosmia, Human Genetics

## Abstract

Short-term, repeated exposure to odors, “olfactory training” (OT), improves olfactory function. Clinically, this works not only for trauma- or disease-related olfactory impairment but also in people with specific anosmia. Androstenone is an odorant for which the frequent occurrence of specific anosmia is already known. It is an odorous steroid derived from testosterone. Besides some people who cannot perceive the odor, it is perceived differently by different individuals in terms of odor quality. These differences in the ability to perceive androstenone as well as in the perception of its quality were previously related to single nucleotide polymorphisms of the human olfactory receptor OR7D4. The current study addressed the question of whether changes in the perception of androstenone in relation to a change in sensitivity following specific OT with that odorant are associated with genetic variations of OR7D4. A total of 335 healthy volunteers participated (206 females, 129 males). All participants underwent tests for normal olfactory function; 103 showed specific anosmia for androstenone. Seventy-seven participants initially unable to perceive androstenone performed OT for an average duration of 8 weeks. Detection thresholds as well as subjective evaluation of odor intensity and pleasantness were measured both before and after OT. Buccal swabs were taken to examine the OR7D4 genotype. The study provided the following major results: (1) Detection thresholds were significantly lower after OT. (2) There was no statistically significant impact of the OR7D4 genotype on the ability to perceive androstenone after OT. In conclusion, it appears that the ability to perceive androstenone can be trained in people with specific anosmia, although OR7D4 polymorphisms were not related to a major change in the sensitivity towards androstenone.

## 1 Introduction

Androstenone (5α-androst-16-en-3-one) is an odorous steroid derived from testosterone (Griffiths and Patterson, 1970, Keller et al., 2007). It can be found in boars, for example in the boar fat or peripheral plasma (Andresen, 1976, Patterson, 1968) but also in humans, for example in saliva (Bird & Gower, 1983), axillary collections, and urine (Araneda and Firestein, 2004, Bird and Gower, 1981). It has been shown that androstenone is variously perceived by different individuals. While some individuals perceive it as a rather pleasant odor (“sweet, floral, vanilla”), others perceive it as more unpleasant (“sweaty, urinous”) (Araneda and Firestein, 2004, Bartoshuk and Beauchamp, 1994, Keller et al., 2007, Stevens and O’Connell, 1996). Furthermore, previous studies have shown that specific anosmia towards the odorant seems to be a widespread phenomenon (Bremner et al., 2003, Labows and Wysocki, 1984, Pause et al., 1999, Wysocki et al., 1989). Specific anosmia is described as “the condition in which a person of otherwise normal olfactory function cannot perceive a particular compound, at a concentration such that its odor is obvious to most other people” (Amoore, 1977). Depending on the selected criteria, definition of anosmia, and test methods, the percentage of people with specific anosmia to androstenone ranges from 1.8 up to 75 percent (Bremner et al., 2003, Triller et al., 2008). In general, men seem to exhibit more often specific anosmia to androstenone than women (Dorries et al., 1989). In addition, when able to perceive the odor, men describe it more often as pleasant while women more often rate it as an unpleasant odor (Griffiths and Patterson, 1970, Wysocki and Gilbert, 1989). The question of why the perception of androstenone differs so widely between individuals has been addressed multiple times. Looking at gender-related differences, a hormonal influence is conceivable. Women tend to perceive androstenone as more pleasant during ovulation than during other phases of the menstrual cycle (Hummel et al., 1991). The percentage of people with specific anosmia to androstenone, especially men, seems to increase with puberty (Dorries et al., 1989). A similar effect has been shown for androstadienone (androsta-4,16-dien-3-one), a closely related odorous steroid, for which a negative correlation between age and odor sensitivity was reported in males (Hummel et al., 2005).

On the other hand, a genetic influence on the perception of androstenone has been suggested in the past (Beets and Theimer, 1970, Wysocki and Beauchamp, 1984). A link between genetic variations of OR7D4 and the perception of androstenone and androstadienone has been shown for the first time in 2007 (Keller et al., 2007). Two non-synonymous single nucleotide polymorphisms (SNPs) resulting in the common variant “WM” seem to severely impair the receptor function in vitro (Keller et al., 2007). Subjects with RT/WM or WM/WM genotype tend to be less sensitive to both steroidal odors and to find both odors more pleasant than RT/RT-individuals (Keller et al., 2007). Similar results were also shown in the perception of cooked meat containing androstenone where the RT/RT group rated the meat less favorable (Lunde et al., 2012).

In addition, previous studies have shown that repeatedly, short-term exposure to androstenone can induce its perception in people with specific anosmia (Stevens & O’Connell, 1995; L. Wang et al., 2004; Wysocki et al., 1989). This could be due to peripheral events like the periodic replacement of olfactory neurons resulting in expansion or selection of olfactory receptors with a higher affinity for androstenone, therefore enabling its perception (Wysocki et al., 1989). Peripheral plasticity is also shown by an increase in the amplitude of the mucosal electro-olfactogram (EOG) and the cerebral olfactory event-related potentials (OERP) after repeated exposure to androstenone in anosmic subjects, with EOG representing peripheral changes and OERP reflecting peripheral and central activities (L. Wang et al., 2004). While it has been shown in the past that the perception of androstenone can be trained in a part of the subjects with specific anosmia, some subjects remain specific anosmic despite being regularly exposed to the odor (Pause et al., 1999, Wysocki et al., 1989).

The current study addressed the question of whether not only the perception but also the ability or inability to learn to smell androstenone is influenced by genetic polymorphisms in OR7D4. It was hypothesized (1) that the perception of androstenone in subjects with specific anosmia could be acquired by regular OT, and (2) that the ability to learn to perceive androstenone as well as the perception in terms of intensity and pleasantness after OT are affected by OR7D4 genotype.

Therefore, subjects with specific anosmia to androstenone were identified and asked to performed OT. OR7D4-genotype was analyzed in a part of the participants. In this study, participants’ perception of three other odors was also tested: benzylsalicylate (BENZ), bacdanol (BAC) and 3-hydroxy-2-methyl-4-pyrone (3H2M4P). These odors were selected since specific anosmia to them is highly prevalent, and they differ in molecular weight. Participants were also asked to fill out three questionnaires before and after OT, and to fill out a diary during OT. This paper focuses primarily on the results regarding androstenone and OR7D4. The remaining results of this study regarding the other odors used, questionnaires, and diaries will be published elsewhere*.* Results regarding the other odors may help understand the results with androstenone regarding the fact that the olfactory training involved all of the odors.

## 2 Material and methods

### 2.1 Participants

A total of 345 volunteers were recruited. The recruitment was done via advertisements at the university, local markets, and online resources, among other channels. Smoking more than 5 cigarettes per week was considered as an exclusion criteria, hence, 10 participants were excluded from the analysis, resulting in a total of 335 participants (206 females, 109 males; age 18–52 years, mean age 27.1 years). The medical history of the subjects was obtained with a structured questionnaire. Only people without current high nicotine consumption (≤5 cigarettes/week) and indicating intact olfactory function were included. Subjects were instructed not to eat, drink or smoke at least 30 min before the appointments to minimize temporary changes in olfactory function.

**Table 1:** Demographic Characteristics of the Participants. Cig.= cigarettes.

N (total)= 335	n	Percent (%)
Gender: male	129	38.5
Gender: female	206	61.5
Native german speakers	288	86.0
Smoking (<5 cig./week)	23	6.9

The study was performed in accordance with the Helsinki Declaration and approved by the ethical committee of the medical faculty of TU Dresden (protocol number EK67022018). All subjects provided written consent after being informed orally and in writing about procedures, aims and potential risks of the study. They received moderate financial compensation for their participation. All tests were performed by the same experimenter.

### 2.2 Initial examination

The initial examination included self-assessment of smell and taste ability. In addition, participants were asked to fill out 3 questionnaires: “WHO 5- Well-being” (Wellbeing measures in primary health care, 1998), “Satisfaction with Life Scale” (Diener et al., 1985), and “Importance of olfaction” (Croy et al., 2009). This was done to gather information about parameters that might influence the ability to become sensitive towards androstenone, like the general interest in odors during daily life.

Normosmia was ascertained with a modified short-version of the Sniffin’ Sticks odor identification test (Burghart, Holm, Germany). Three odors were presented to each subject for a few seconds. If necessary, the procedure was repeated once. Participants were asked to identify each odor from a list of 4 descriptors. All three odors had to be identified correctly to determine normosmia. If not, testing with Sniffin’ Sticks was extended to ensure normosmia. Hyposmic participants were excluded from participation in the study. For threshold measurements, androstenone (5α-androst-16-en-3-one) was serially diluted in propylene glycol to obtain concentrations of 50 µg/g, 5 µg/g, 0.5 µg/g, 0.05 µg/g, 0.005 µg/g. A 6th dilution stage (0.0005 µg/g) was added during a later stage of the experiments in order to prevent ceiling effects of the measurements. Solutions were stored in brown glass bottles and prepared at regular intervals (every two to three months) to maintain stable concentrations of the odors. During the examination of odor threshold, participants were blindfolded to prevent visual identification of the odor-containing bottles. The experimenter presented three bottles to each subject in an ad hoc randomized order, two containing only the solvent and one containing the odor at a certain concentration. The participant was asked to pick out which of the 3 bottles smelled differently. Presentation of triplets occurred with an interval of a few seconds to avoid adaptation. Starting in ascending order of the odor dilutions, subjects had to correctly discern the odorant in two successive trials. Based on the staircase-method (Ehrenstein & Ehrenstein, 1999), concentrations were then presented in an alternately descending and ascending way until three turning points were made. The mean of these three concentrations was used as the threshold estimate. Only subjects who could not identify androstenone at the highest concentration or had a threshold between the first two dilution steps were considered specific anosmic, and therefore asked to participate in OT. Afterward, subjects were asked to sniff at the bottle containing the highest concentration of each odor and rate the perceived intensity on a scale from 0 to 10 (0 = “nothing”; 10 = “extremely intense”), and pleasantness on a scale from −5 to + 5 (-5 = “not at all pleasant”, +5 = “extremely pleasant”). Participants were also asked to describe the odor quality of androstenone in their own words and describe it based on the selection of different descriptors.

### 2.3 Olfactory training

Subjects with specific anosmia to androstenone were asked to participate in an olfactory training. In total, 77 participants with specific anosmia to androstenone completed OT. Participants were divided into three groups, with the largest group (Group 1, n = 68; 36 females, 32 males; mean age 26.5 years) training with androstenone for 60.6 days on average. In addition, two smaller groups were established. Subjects in the second group (Group 2, n = 5; 2 females, 3 males; mean age 24.8 years) showed specific anosmia for androstenone but did the OT with other odors (BAC, BENZ, and 3H2M4P, OT duration 67.2 days on avarage) to investigate whether androstenone is an essential component of OT to improve its perception. The third group (Group 3, n = 4; 3 women, 1 man; mean age 36 years, OT duration 55.3 days on avarage) stated to not perceive androstenone when presented at the highest concentration but had thresholds in the “osmic” range. In this group, OT was performed with all 4 odors to investigate if perception of androstenone could be improved.

Participants were instructed to perform OT twice a day, in the morning and in the evening. Each subject received brown glass bottles containing a cotton ball soaked with the respective odor. For training, 1 ml of androstenone (50 µg/g) was used. Details regarding the other three odors, for example the amount used for OT, are published in a separate paper. A training session included the following steps, and the associated instructions were given to the participants:

- Opening a smelling bottle

- 20 s of sniffing the odor

- Closing the bottle

and was carried out with each smelling bottle. This procedure was to be repeated once so that a full training session included smelling each scent twice and took about three minutes. In order to be able to better reconstruct the course of the olfactory training in retrospect participants were given an olfactory diary, where they were instructed to evaluate the perceived odor intensity of each odor on a scale from 0 to 10 and to document any peculiarities once a week on the same day. The olfactory diary was subsequently used to ensure that training was carried out regularly.

### 2.4 Final examination

After completing OT, participants were examined as in the initial examination: they were asked to fill out the same three questionnaires, odor thresholds were measured using the same method, and participants were again asked to rate the perceived intensity and pleasantness of the highest concentration of the odors, as well as to describe androstenone smell.

### 2.5 Mucosa sampling and analysis of the OR7D4 genotype

Before and after OT, buccal swabs were used to collect samples of subject’s saliva and mucosa. To prevent contamination, the examiner wore gloves. Collected samples were stored at −30°C (-20°C?) and on dry ice. Genomic DNA was extracted from buccal swabs using the Qiagen QIAamp DNA Investigator kit (Qiagen Cat# 56504), with 2h20min of Proteinase K digestion and elution in 30 μl of buffer. Eluted DNA concentration was measured using Qubit. 5 ng of DNA was used in 10 μL PCR reaction containing 1x PCR buffer (Qiagen #203205), 0.25 mM dNTPs, 0.5 μM 5‘ primer (CAGCAGACACAACAGCTAC), 0.5 μM 3’ primer (GTTCTGAGGCCCTGATTTG), 0.05 U Hot Start Taq polymerase (Qiagen #203205). DNA amplification was performed using the conditions: initial denaturation at 95°C for 15 min, followed by 35 cycles of 94°C for 30 sec, 55°C for 30 sec, 72°C for 30 sec, and final extension at 72°C for 5 min. Amplification was verified in gel electrophoresis using 1 μl of PCR reaction. The remaining PCR product was mixed with water to a final volume of 35 μl and purified using Sephacryl S-400. 2 μl of purified PCR product was used in Sanger Sequencing with primer GAACCTGCTCATCATTCTG.

### 2.6 Data analysis

For statistical analysis SPSS Statistics Standard (Statistic package for the social sciences; SPSS Inc., Chicago, IL, USA) was used. Statistical analyses of independent samples were performed using *t-test* or Mann-Whitney *U* test. Pearson statistics were used for correlation analyses. Significance level was set at 0.05.

## 3 Results

### 3.1 Perception of androstenone

103 out of 335 participants (30.75 %) were classified as anosmic for androstenone, being 57 of them women (or 28 % among female participants) and 46 men (36 % among male participants). There was no significant impact of gender or age on the ability to perceive androstenone (P_Gender_=0.12; P_Age_=0.81).

On average, subjects able to perceive androstenone rated the odor as unpleasant (M = −0.9, SD = 2.06). Most used descriptors were “harsh”, “sweaty” and “sharp”. Some frequently used descriptors associated with pleasant perception were “sweet”, “floral” and “woody”.

While there were no significant gender-related differences observed for androstenone threshold and intensity rating, the odor pleasantness was rated significantly lower by women than by men (n = 335; M_Female_ = −0.85; SD = 1.80, M_Men_ = −0.24; SD = 1.96; P = 0.002, Mann-Whitney *U* test).

**Fig. 1:**
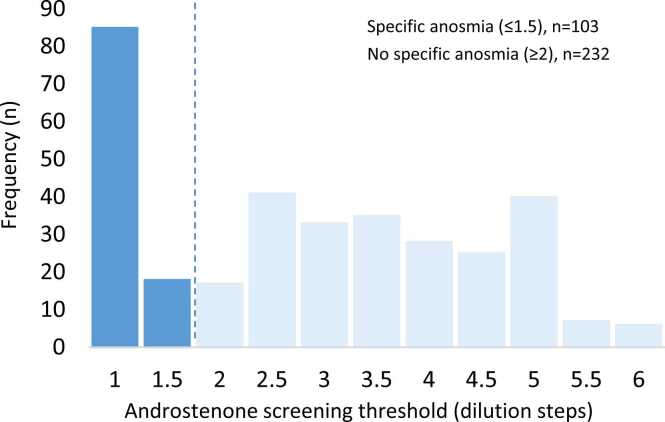
Frequency distribution of olfactory thresholds for androstenone.

### 3.2 Olfactory training results

49 out of 68 participants (72 %) in training group 1 were able to detect androstenone after OT according to the measured threshold, and the mean threshold score was significantly lower after OT than before (M_before_ = 1.01; SD = 0.08, M_after_ = 2.86; SD = 1.44; P < 0.001, Mann-Whitney *U* test). Participants also rated the intensity of androstenone significantly higher after OT (M_before_ = 0.99; SD = 1.06, M_after_ = 2.97; SD = 2.02; P < 0.001). Even though there was no significant difference in ratings of pleasantness according to the mean values, pleasantness ratings after OT were more widely distributed (M_before_ = 0.15; SD = 1.02, M_after_ = 0.09; SD = 1.62; P = 0.63).

In group 2 (n = 5) the androstenone threshold of four subjects remained the same after OT with BENZ, BAC and 3H2M4P but without androstenone. The threshold of one subject lowered 0.5 points but was still considered anosmic to androstenone. There was no statistically significant difference between mean thresholds for androstenone before and after OT (M_before_ = 1.0; SD = 0, M_after_ = 1.10; SD = 0.2; P = 0.32). There was no significant difference in androstenone thresholds for group 3 as well (n = 4; M_before_ = 2.25; SD = 0.25, M_after_ = 3.38; SD = 1.19; P = 0.18). No significant change in ratings of intensity or pleasantness was observed in either of the two groups, even though the main intensity rating for androstenone was higher after OT in group 3 (M_before_: 0.25, SD: 0.43, M_after_: 4.0, SD: 0.71).

For training group 1, there were no statistically significant gender-related differences in odor thresholds before and after OT. There was a significant increase in intensity ratings documented in the “OT-diaries”. For statistical analyses the values from weeks 1 and 8 were compared (n = 48; M_week1_ = 2.49; SD = 2.04, M_week8_ = 3.92; SD = 2.08; P < 0.001).

**Fig. 2:**
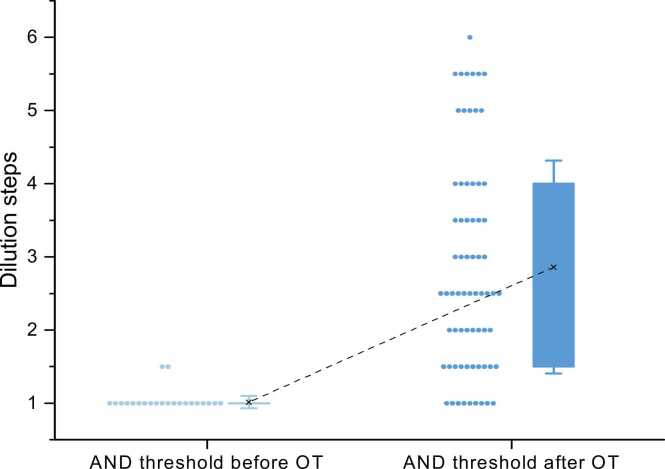
Thresholds for androstenone before and after olfactory training.

### 3.3 OR7D4 polymorphism analysis

OR7D4 genotype was obtained for 137 (61 males, 76 females) participants, 88 of them being specific anosmic for androstenone and 49 being able to perceive androstenone. OR7D4 variants frequency among genotyped participants was 95 RT/RT (69 %), 33 RT/WM (24 %), and 9 WM/WM (7 %). There was no significant difference between the OR7D4-genotype of subjects with or without specific anosmia towards androstenone. There was also no significant impact of gender on the OR7D4 genotype.

Out of the 49 subjects being able to perceive androstenone, 38 showed RT/RT-genotype (78 %), 9 RT/WM (18 %), and 2 WM/WM (4 %). Because of the small number of WM/WM individuals, subjects with at least one WM allele were grouped for statistical analyses, leading to a total of 11 participants in the RT/WM / WM/WM group. There was no significant difference in odor threshold or perceived intensity of androstenone between RT/RT and RT/WM / WM/WM groups. Participants with at least one WM- allele rated androstenone significantly more “pleasant” than RT/RT subjects (M_RT/RT_ = −1.37; SD = 1.86, M_WM-allele_ = 0.18; SD = 1.54; P = 0.016). Out of the 88 subjects with specific anosmia to androstenone, 57 had RT/RT genotype (65 %), 24 RT/WM (27 %), and 7 WM/WM (8 %).

**Fig. 3:**
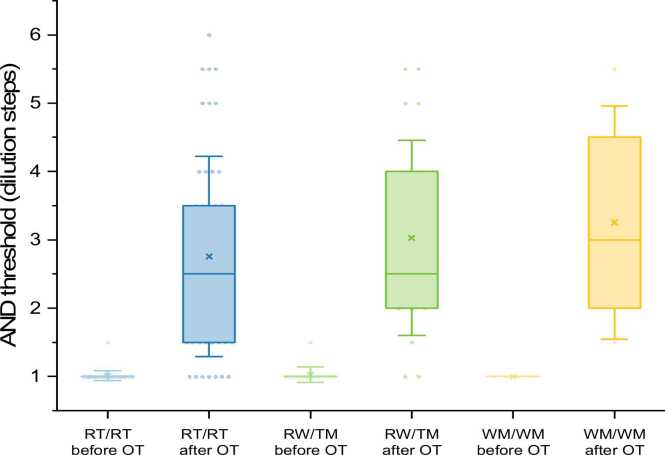
Androstenone thresholds before and after OT depending on the OR7D4-genotype.

**Fig. 4:**
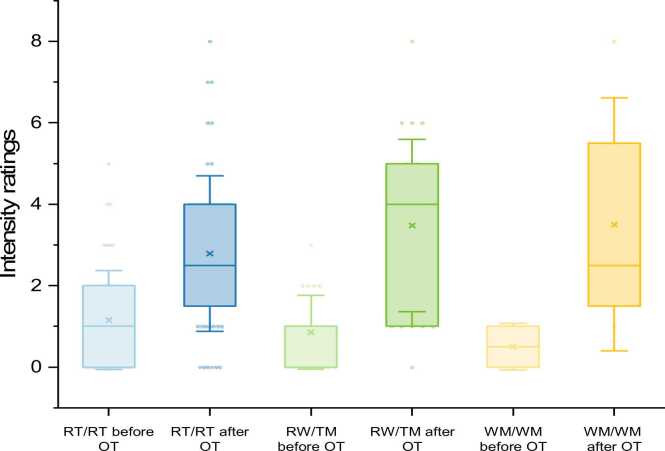
Intensity ratings for androstenone before and after OT depending on the OR7D4-genotype.

The OR7D4 genotype of all 68 participants in training group 1 was analyzed. 49 subjects were able to perceive androstenone after OT, from which 30 had the RT/RT-genotype (61.2 %), 16 RT/WM (32.7 %), and 3 WM/WM (6.1 %). 19 subjects were unable to perceive androstenone after OT, from which 15 had the RT/RT-genotype (79 %), 3 RT/WM (16 %), and 1 WM/WM (5 %). Differences in OR7D4 variant frequencies were not statistically significant (p = 0.35).

There was no significant difference in androstenone thresholds after OT in subjects with specific anosmia and different OR7D4 genotypes. Therefore, there was no significant impact of OR7D4-genotype on the ability to learn to perceive androstenone by OT. There was also no significant difference in intensity or pleasantness rating between RT/RT subjects and individuals with a WM- allele after OT. In training group 1 (n = 68) there were 45 RT/RT, 19 RT/WM, and 4 WM/WM subjects. The mean thresholds and perceived intensity in RT/RT and RT/WM individuals were significantly higher after OT than before (p < 0.001). No statistical analysis was performed for WM/WM subjects due to the low number of individuals.

## 4 Discussion

The results of the present study confirm the assumption of specific anosmia to androstenone being a widespread phenomenon. This applied to around a third of the study participants, which is in line with previous studies (Dorries et al., 1989, Wysocki and Gilbert, 1989). Also in accordance with former findings the percentage of male participants showing specific anosmia to androstenone was higher than in females (Dorries et al., 1989, Griffiths and Patterson, 1970, Wysocki and Gilbert, 1989). However, other studies reported a lower percentage of subjects with specific anosmia to androstenone (Beets and Theimer, 1970, Pause et al., 1999). These differences may be due to different odor delivery methods, diluents, data interpretation and criteria for defining specific anosmia since it has been shown that these may affect the measured thresholds (Doty et al., 1995, Frasnelli et al., 2002, Pierce et al., 1996). According to Bremner et al. 2003 the estimated percentage of specific anosmia to androstenone in young adults is 1.8–5.96 %, with the authors suggesting that in previous studies most subjects considered as non-detectors are in fact specific hyposmics. In the present study the defined threshold for specific anosmia was more liberal and the threshold measurement shorter compared to Bremner et al. 2003, possibly resulting in Type II error and falsely labeling some hyposmic detectors as non-detectors. In addition, it is known that androstenone can also stimulate the trigeminal system in some people, leading to the perception of a “sharp” sensation (Boyle et al., 2006). Participants therefore may be able to identify the odor correctly in higher concentrations, despite being specific anosmic (Boyle et al., 2006, Croy et al., 2016).

Like in previous studies, the qualitative perception of androstenone varied among participants. While there were no sex-related differences in androstenone threshold or intensity ratings, the odor was rated as significantly less unpleasant by men, as demonstrated in previous studies (Griffiths and Patterson, 1970, Wysocki and Gilbert, 1989). Hormonal influence on the perception of androstenone seems conceivable according to our results. Still, the evidence in this study is limited since taking oral contraception was no exclusion criteria and for technical reasons the phase of menstrual cycle was not taken into account.

Sensitivity to androstenone was acquired in 72 % of participants from group 1 after OT, confirming previous findings that regular exposure to androstenone changes its perception in most but not all participants (Mainland et al., 2002, Möller et al., 1999, Pause et al., 1999; L. Wang et al., 2004; Wysocki et al., 1989). However, the thresholds for androstenone of all subjects in group 2 (who did not train with androstenone) remained in the anosmic range. Therefore, it can be assumed that, at least in the case of androstenone, the odor to which an individual is specific anosmic, needs to be a component in the OT whereas OT with other odors has little or no impact on its perception. Still, as the group size of the second training group in the present study comprises only 5 people, the resulting limited statistical power should be considered. Hence, this should be re-evaluated in future studies in larger groups. Also, looking at the results of training group 3, individual perception of androstenone can be improved by OT. The missing perception of androstenone in subjects with thresholds in the “osmic” range highlights the importance of a more exact screening method.

In this study, the majority of participants had RT/RT and RT/WM genotypes, whereas WM/WM genotype was found in less than 10 %. This is in line with results from previous studies, which showed that WM/WM genotype is rare in the population (Hornung et al., 2018, Keller et al., 2007) and with the 1000 genome project data (see: *OR7D4 Olfactory Receptor gene symbol*, o. J.). Results of the present study do not confirm a significant difference in OR7D4 genotype between subjects with or without specific anosmia to androstenone. Nonetheless, the percentage of RT/RT subjects was slightly higher for the “detectors” whereas more RT/WM and WM/WM subjects were found in the ”non-detection” group. The lack of significance could be due to the unequal group size of detectors and non-detectors or a small number of participants in general – although the overall group size was larger than 300. However, a significant impact of OR7D4 genotype on the ability to perceive androstenone has been shown in previous studies (Knaapila et al., 2012), and with fewer subjects (Li et al., 2022, Lunde et al., 2012).

In the present study, a significant impact of OR7D4 genotype on androstenone perception by normosmic subjects was only found for pleasantness but not for threshold or intensity. In addition, OR7D4 genotype had no significant impact on the outcome of OT in subjects with specific anosmia to androstenone. Furthermore, differences in androstenone thresholds, intensity, and pleasantness after OT did not significantly associate with OR7D4 genotype. In contrast to the assumptions of previous studies (Lunde et al., 2012), RT/RT subjects were less likely to be able to perceive AND after OT than RT/WM and WM/WM subjects. Furthermore, subjects with at least one WM-allele achieved better training results on average. However, with these findings not being statistically significant we point out the need for sufficiently large sample sizes. Altogether, our results could not replicate previous studies where OR7D4 genotype was shown to have a significant impact on the perception of androstenone. The present findings are more in line with Hornung et al., who found no significant difference in thresholds, intensity, and pleasantness ratings of androstadienone between homozygous carriers of the RT-allele and heterozygous carriers (Hornung et al., 2018).

Altogether, no significant impact of the OR7D4 genotype on the olfactory training results with androstenone could be demonstrated. Contrary to the assumptions from earlier studies (Lunde et al., 2012), participants with the RT/WM or WM/WM genotype were even more frequently and more strongly sensitized to androstenone. Overall, the results of the present study suggest that the perception and learnability of androstenone cannot be explained by single nucleotide polymorphisms of OR7D4 alone. In particular, other factors seem to play a role in olfactory training. This is further discussed in our follow-up paper, were we also present the results of olfactory training with benzylsalicylate, bacdanol and 3-hydroxy-2-methyl-4-pyrone. In summary, among many factors, the following mechanisms can be considered: increased olfactory receptor expression or changes in the olfactory receptor structure (H. W. Wang et al., 1993; L. Wang et al., 2004), the presence of other androstenone-sensitive receptors (Keller et al., 2007), changes in the olfactory mucus (Croy et al., 2015, Zou et al., 2020), top down effects from the central nervous system (Cavazzana et al., 2018), as well as gender, age, or environmental influences (Bratman et al., 2024).

In conclusion, the results of the present study confirm previous findings that the ability to perceive androstenone in subjects with specific anosmia can be acquired by regular exposure to the odorant. Therefore hypothesis 1 was confirmed. However, the overall results do not support the idea of a significant impact of the hOR7D4 genotype on the perception or the ability to improve androstenone sensitivity by OT. Hypothesis 2 could not be confirmed. Thus, it is likely that OR7D4 does not alone fully account for the perception of androstenone. Future studies are needed to investigate other influencing factors such as changes in the olfactory mucus, individual gene expression mechanisms, or the influence of human olfactory receptors other than OR7D4.

### 4.1 Limitations

Our study has the following limitations:

The olfactory training took place without supervision and therefore on the participants' own responsibility. Reliability in terms of regularity and an accurate following of the instructions could therefore not be fully verified. Despite a fairly large number of individuals tested (n = 335), only a few individuals with the OR7D4-WM/WM genotype could be found. Also, the number of participants in training groups 2 and 3 was limited to a small number. Therefore, we suggest that this data needs confirmation in a larger study group. In addition, re-test accuracy has not been tested in the present study. Former studies have shown that the detection threshold values are more reliable than, for example, recognition threshold values; whereby those based upon a single ascending presentation series are much less reliable than those based upon a staircase procedure (Doty et al., 1995, Hummel et al., 1997). Also, reliability seems to be dependent from test-length (Doty et al., 1995). While there are few data on the re-test accuracy of androstenone thresholds as they have been obtained in the present study, the results can be considered reliable as a standard staircase procedure had been used, although it had been abbreviated for the sake of time. The reliability was further increased because this highly standardized procedure was always carried out by the same investigator. In addition, most subjects initially anosmic to androstenone described its smell as unpleasant, musky or sweaty after the olfactory training. Based on the olfactory diaries, a slow increase in the ability to perceive androstenone could be traced over several weeks. Still, further studies are needed to investigate the re-test accuracy of androstenone thresholds over a short period of time.

## Funding

The study was supported by a grant from the Volkswagenstiftung to TH (project PERCEPTRONICS, Az 9B396).

## CRediT authorship contribution statement

**Antonia Gillmeister:** Writing – original draft, Project administration, Methodology, Investigation, Formal analysis, Data curation, Conceptualization. **Maira Harume Nagai:** Writing – original draft, Project administration, Methodology, Investigation, Formal analysis, Data curation. **Priyanka Meesa:** Writing – review & editing, Methodology, Data curation. **Christian Margot:** Writing – review & editing, Methodology. **Hiroaki Matsunami:** Writing – review & editing, Supervision, Project administration, Methodology, Funding acquisition, Formal analysis, Data curation, Conceptualization. **Thomas Hummel:** Writing – review & editing, Validation, Supervision, Project administration, Funding acquisition, Conceptualization.

## Declaration of Competing

During the last 3 years TH collaborated with the following companies: Sony, Tokyo, Japan; Smell and Taste Lab, Geneva, Switzerland; Takasago, Paris, France; Cyrano, Delray Beach, FL, USA; Cynexo, Trieste, Italy; Sentosphere, Paris, France; NOAR, Sao Paulo, Brazil H.M. has received royalties from Chemcom, research grants from Givaudan, and consultant fees from Kao.AG, MN, PM an CM have nothing to declare.
